# Predictive Value of the Interaction between CEA and Hemoglobin in Neoadjuvant CCRT Outcomes in Rectal Cancer Patients

**DOI:** 10.3390/jcm12247690

**Published:** 2023-12-14

**Authors:** Yi-Hsuan Lai, Yu-Tien Chang, Yu-Jia Chang, Jo-Ting Tsai, Ming-Hsien Li, Jang-Chun Lin

**Affiliations:** 1 Department of Radiation Oncology, Shuang Ho Hospital, Taipei Medical University, New Taipei City 235041, Taiwan; 21266@s.tmu.edu.tw (Y.-H.L.); 10576@s.tmu.edu.tw (J.-T.T.); 09112@s.tmu.edu.tw (M.-H.L.); 2 School of Public Health, National Defense Medical Center, Taipei 114201, Taiwan; greengarden@mail.ndmctsgh.edu.tw; 3 Graduate Institute of Clinical Medicine, College of Medicine, Taipei Medical University, Taipei 110301, Taiwan; r5424012@tmu.edu.tw; 4 Department of Radiology, School of Medicine, College of Medicine, Taipei Medical University, Taipei 110301, Taiwan

**Keywords:** LARC, CCRT, CEA, Hb, treatment response, interaction, TRG, predictive biomarkers

## Abstract

The adoption of neoadjuvant concurrent chemoradiotherapy (CCRT) has reshaped the therapeutic landscape, but response prediction remains challenging. This study investigates the interaction between pre-CCRT carcinoembryonic antigen (CEA) and post-CCRT hemoglobin (Hb) levels in predicting the response of locally advanced rectal cancer (LARC) to CCRT. Retrospective data from 93 rectal cancer patients receiving neoadjuvant CCRT were analyzed. Univariate analyses assessed clinical factors associated with tumor regression grade (TRG) and T-stage outcomes. Machine learning identified predictive biomarkers. Interaction effects between CEA and Hb were explored through subgroup analyses. Post-CCRT Hb varied between pre-CCRT CEA groups. The interaction between pre-CCRT CEA and post-CCRT Hb influenced TRG. Males with normal pre-CCRT CEA and anemia showed better treatment responses. Females with elevated pre-CCRT CEA and post-CCRT anemia exhibited poorer responses. The interaction effect between them was significant, indicating that their relationship with TRG was not additive. Inflammatory biomarkers, WBC, neutrophil count, and post-CCRT platelet level correlated with CCRT response. Contrasting with previous findings, anemia was a predictor of better treatment response in males with normal pre-CCRT CEA. The interaction between pre-CCRT CEA and post-CCRT Hb levels predicts the response of LARC to CCRT. CEA, Hb, and sex should be considered when assessing treatment response. Inflammatory biomarkers contribute to response prediction. Understanding these complex relationships can enhance personalized treatment approaches in rectal cancer patients.

## 1. Introduction

Colorectal cancer (CRC) stands as a prevalent form of malignancy on the global scale. Rectal cancer makes up about one third of its overall incidence. Annually, this subtype is diagnosed in over 100,000 individuals across the world. There is a notable trend in CRC oncology toward diagnosing the disease at a younger age and at more advanced stages [1,2]. The management strategy for locally advanced rectal cancer (LARC) has undergone substantial transformation, especially in the application of radiotherapy. Initially, adjuvant concurrent chemoradiotherapy (CCRT) was the standard approach after surgical resection, but in recent years, the paradigm has shifted toward neoadjuvant CCRT as the preferred therapeutic approach [3]. Extensive evidence supports the efficacy of upfront CCRT prior to surgery, as it has demonstrated enhanced local control and reduced toxicity during both immediate and prolonged observation [4,5]. This neoadjuvant strategy offers several benefits, including downsizing of tumors, increasing the likelihood of complete resection, and potential sphincter preservation in selected cases. For tumor downsizing, achieving a pathological complete response (pCR) has been linked to better five-year disease-free survival (DFS) and overall survival (OS). Furthermore, when specifically considering the therapeutic effectiveness of the primary tumor, achieving a complete response (ypT0) also brings a survival benefit [6]. Unfortunately, not all patients respond to CCRT, and the pCR rate remains low (10–25%) [7,8]. Additional systemic chemotherapy after CCRT before surgery can be administered, but this increases the risk of toxicity. A better tumor response after CCRT results in a relatively lower recurrence rate [9]. Therefore, it is essential to predict the response to neoadjuvant CCRT.

Laboratory tests offer valuable insights into a wide range of biomarkers, including tumor markers, inflammatory markers, genetic markers, and immunological markers, which are relevant for predicting treatment response in LARC [10]. However, certain biomarker tests may be costly or challenging to obtain. Hence, concentrating on commonly available laboratory tests to identify potential clues for risk stratification in rectal cancer may yield significant benefits for the clinical situation. Extensive research has shown strong correlations between rectal cancer and commonly observed tumor markers such as carcinoembryonic antigen (CEA) and carbohydrate antigen 19-9 (CA-19-9) [11]. The CEA level is a widely recognized indicator of disease activity in cases of CRC. Elevated CEA, both before surgery and throughout the monitoring phase, is associated with an increased likelihood of tumor recurrence and poorer overall OS. A CEA threshold of 5 ng/mL is commonly employed to distinguish between normal and elevated levels. Monitoring the trend of CEA levels, whether they decrease or increase, can provide valuable information about the severity and progression of the disease [12,13].

Among CRC patients, the occurrence of anemia varies from 22.8% to 35% [14,15]. LARC patients often have low hemoglobin (Hb). Several factors can cause anemia, one of which involves the presence of hematochezia, which is characterized by the passage of blood through the rectum and serves as a prominent symptom of rectal cancer. The chronic blood loss associated with rectal cancer leads to the development of anemia. The adverse effects of adjuvant chemotherapy regimens can also contribute to anemia in patients with CRC. As a result, anemia can substantially influence the treatment results for patients with CRC.

On the other hand, anemia decreases the oxygen supply, fostering a hypoxic tumor microenvironment that enhances tumor growth and therapeutic resistance. Insufficient oxygen compromises radiation therapy by limiting reactive oxygen species generation and impairing cancer cell damage and radiation-induced cell death [16]. Anemia serves as an independent prognostic indicator of inadequate local control in rectal cancer [17,18]. Considering both overall survival and survival specific to LARC, older individuals with preexisting anemia have notably lower survival rates [19]. Given that anemia is a frequent adverse effect of chemotherapy agents such as oxaliplatin, fluorouracil, and capecitabine, which are commonly used in neoadjuvant CCRT regimens for LARC, the incidence of anemia may increase following CCRT. Clinical management of anemia in rectal cancer involves nutritional support, iron supplementation for suspected iron deficiency, and blood transfusion for severe anemia or acute blood loss. The potential benefits of preoperative correction of anemia and whether anemia can be used as a biomarker to predict the effectiveness of neoadjuvant CCRT remain uncertain.

Recent studies have suggested that pretreatment laboratory values, such as CEA and Hb, might also help predict the treatment response [20,21]. While the independent effects of CEA and Hb on CCRT outcomes in rectal cancer have been studied, the potential interaction between these two factors has not been fully explored. Understanding the interaction between these two laboratory values could provide insight into their combined effects on treatment response and could assist in formulating more efficient therapeutic approaches for individuals with rectal cancer. In this study, our objective was to examine the potential interaction between CEA levels and Hb levels and their effects on CCRT outcomes in patients with rectal cancer. Specifically, we intended to utilize these findings to stratify the risk of LARC patients and enhance the development of personalized precision medicine, thereby enabling the formulation of tailored treatment strategies for individuals with LARC.

## 2. Materials and Methods

### 2.1. Patient Eligibility

This study was a retrospective analysis of medical records of patients diagnosed with rectal cancer who underwent neoadjuvant CCRT at Taipei Medical University—Shuang Ho Hospital between 2017/5 and 2022/02. The institutional review board of Taipei Medical University—Shuang Ho Hospital granted approval for this study. Inclusion criteria were as follows: (1) histologically confirmed adenocarcinoma of the rectum; (2) received neoadjuvant CCRT prior to surgical resection; (3) available pretreatment serum CEA level; (4) age 20–90 years; (5) no previous treatment for rectal cancer; (6) complete medical records available for analysis; and (7) having undergone surgery at Shuang Ho Hospital. Patients who did not meet all of the mentioned criteria were excluded from the study. We extracted the following data from electronic medical records: age at diagnosis, sex, pre- and posttreatment CEA levels, Hb level, complete blood count, biochemical results, TNM stage, CCRT regimen, surgical procedures, pathologic tumor and nodal stage, and follow-up information. We divided patients into subgroups by pre-CCRT CEA level, Hb level, and sex. CEA was classified as elevated if it was greater than 5 ng/mL, and anemia was characterized as Hb below 12 g/dL for women and below 13 g/dL for men.

### 2.2. Treatment Modality

The radiotherapy technique employed in this study involved treatment planning using either Pinnacle version 14.0 software for intensity-modulated radiation therapy (IMRT), volumetric modulated arc therapy (VMAT), or tomotherapy with the Hi-ART planning station version 5.1.1.6. Patients were positioned in the prone position for radiotherapy delivery while they had a full bladder to aid in sparing organs at risk. In selected cases, MRI was performed, and MRI fusion was used for image guidance, enabling precise target localization and contouring based on the RTOG consensus guidelines for rectal cancer [22]. The clinical target volume (CTV) for treating rectal cancer encompassed various areas, including the internal iliac, perirectal, and presacral regions. The CTV extended 2 cm below the visible disease in the lower pelvis, covering the complete mesorectum down to the pelvic floor. The mid-pelvic CTV included the rectum, its associated mesentery, and the internal iliac region. In the upper pelvis, the CTV extended either to the rectosigmoid junction or up to 2 cm above the uppermost point of the disease. For rectal carcinomas involving adjacent organs, the external iliac region was also included, with a recommended margin of 7–8 mm around the iliac vessels. Planning target volumes (PTVs) had a margin of 0.5 to 0.7 cm. Treatment was delivered using a 10 MV beam energy, ensuring optimal dose coverage of the primary tumor (50 Gy/25 fractions) and pelvic lymph nodes (45 Gy/25 fractions). CBCT was optionally employed for treatment verification, ensuring accurate patient positioning, while comprehensive quality assurance measures guaranteed treatment plan accuracy and patient safety. Among the 93 patients included in the study, 50 received neoadjuvant CCRT with a fluoropyrimidine-based regimen, specifically 5-fluorouracil (5-FU) and leucovorin. Additionally, 36 patients were prescribed a biweekly schedule of folinic acid, fluorouracil, and oxaliplatin (FOLFOX) along with irradiation. Following the completion of radiation treatment, 49 patients received oral chemotherapy with tegafur. All patients underwent surgery following CCRT, and we ensured a time gap between completing radiotherapy and the surgical procedure. Understanding that the optimal response to radiotherapy typically occurs after 6 to 10 weeks [23], our study observed an average interval of approximately 55.8 days, with a standard deviation of around 13.6 days.

### 2.3. Observational Index

The main focus of this study was to assess how the response to CCRT is influenced by the levels of CEA and Hb. Tumor regression grade (TRG) and T-downstaging were used to assess the response to CCRT. Blood examinations were performed on the patients at least twice. The initial blood samples were collected before the commencement of the first chemotherapy cycle, typically within a week, in an outpatient setting. If patients required hospitalization for chemotherapy administration, blood examinations were conducted on the first day of admission. For post-CCRT data, blood samples were obtained during the initial follow-up after the completion of the last cycle of chemotherapy and before the surgical intervention. The TRG, which was graded by assessing the extent of histological tumor regression in the surgical specimen, is a critical prognostic factor that assesses tumor response after CCRT. TRG can be a valuable and autonomous prognostic factor for long-term DFS in individuals diagnosed with rectal cancer who undergo CCRT [24,25,26]. Our study utilized the American Joint Committee of Cancer and College of American Pathologists (AJCC) four-category system for TRG classification, which is a commonly used TRG systems [27]. To investigate the impact of pre-CCRT CEA and Hb levels on TRG and their interaction effects, TRG scores were assigned to surgical specimens examined by our pathologist: no residual tumor, grade 0; rare small group of cancer cells or single cell, grade 1; residual mass with evidence of regression, but more than rare small cancer cell groups or single cancer cells, grade 2; no evidence of tumor regression in extensive residual tumor, grade 3. In addition, we performed patient grouping based on their pre-CCRT CEA and Hb levels to identify a subgroup that might benefit more from neoadjuvant CCRT.

### 2.4. Statistical Analysis

Patient characteristics were summarized using descriptive statistics. Random forest, a machine learning method, was employed to identify potential variables linked to tumor response. To handle missing data, deletion was performed, and the analysis utilized all available variables. The dataset was divided into a training set, which consisted of 70% of the data, and a testing set, which comprised the remaining 30% of the data. This division facilitated the assessment of the model’s accuracy. The associations of CEA level and Hb level with TRG was evaluated. We plotted the interaction of clinical variables of interest with tumor regression grades using R software version 4.2.2 and the “cat_plot” function from the “interactions” package. For subgroup analysis, two-way ANOVA was performed to validate the interaction between the CEA level and Hb level using SPSS (version 25.0). Statistical significance was defined as *p* < 0.05.

## 3. Results

### 3.1. Patients’ Characteristics

Ninety-three individuals diagnosed with rectal cancer and receiving neoadjuvant CCRT, with accessible pretreatment serum CEA data, constituted the study cohort. The clinical and demographic characteristics are presented in Table 1. The patients were divided into two groups based on their pre-CCRT CEA levels: the pre-CCRT CEA < 5 group (n = 44, 47.3%) and the pre-CCRT CEA ≥ 5 group (n = 49, 52.7%). No statistically significant differences were detected between the two groups regarding age, sex, pre-CCRT T stage, pre-CCRT N stage, pre-CCRT total stage, body weight, underlying diseases, chemotherapy regimen, tobacco use, or oral chemotherapy after CCRT.

### 3.2. Clinical Factors Correlated with Treatment Response

We examined several well-known clinical predictors of rectal cancer treatment response, including CEA, Hb, white blood cell (WBC) count, neutrophil/lymphocyte ratio (N/L ratio), and platelet/lymphocyte ratio (P/L ratio). Table 2 and Table 3 presents the outcomes of the conducted univariate analysis. Table 2 represents the analysis of the association between clinical factors and TRG in resected pathological tissue. A favorable response was characterized as TRG = 0 or 1, while an unfavorable response was indicated by TRG = 2 or 3. Significant associations were found between serum WBC counts before and after CCRT and TRG. Similarly, a consistent trend was observed in neutrophil counts. Lower WBC and neutrophil counts were indicative of a better tumor response. A higher post-CCRT platelet count was associated with a poorer tumor regression grade. Table 3 indicates the relationships between the T-stage outcome and various clinical factors. We defined complete response as the absence of any remaining tumor, good response as a pathological T stage lower than the pre-CCRT clinical T stage, stable disease as no change in the T stage before and after CCRT, and disease progression as an aggravation of the primary tumor after CCRT. The results revealed a significant association between pre-CCRT CEA levels and T-stage outcome, with CEA being higher in patients with more advanced T stages. Moreover, pre-CCRT and post-CCRT WBC and neutrophil counts showed significant associations with T-stage outcome, with lower counts in the complete response group than the other groups. The levels of lymphocytes were notably higher in the disease progression group than in the other groups.

### 3.3. Clinical Factors’ Interaction Effects on TRG

**Figure 1:**
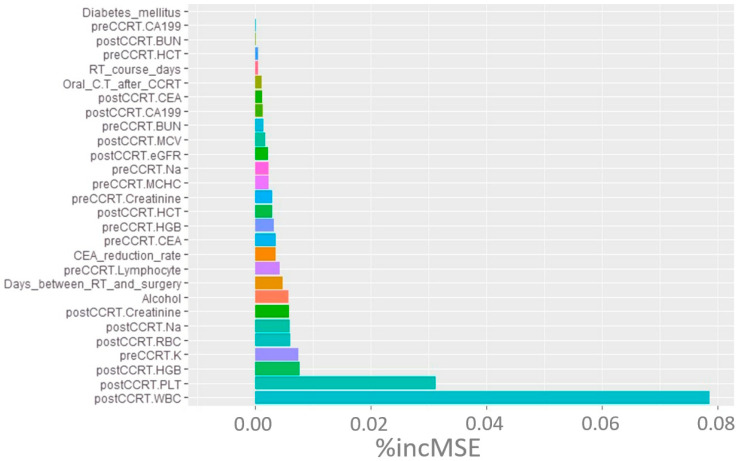
Random forest machine learning analysis of clinical markers contributing to TRG. Abbreviations: TRG, tumor regression grade; % incMSE, percentage increase in mean squared error; CCRT, concurrent chemoradiotherapy; CEA, carcinoembryonic antigen; HGB, hemoglobin; WBC, white blood cells; PLT, platelet; RBC, red blood cell.

Based on the machine learning analysis depicted in Figure 1, post-CCRT WBC count, platelet count, and Hb emerged as the three factors most strongly correlated with the treatment response of rectal cancer. To explore potential correlations or interactions, we evaluated the clinical factors after CCRT in the two groups divided around the median CEA level (Table 4). The findings indicate that individuals with pre-CCRT CEA ≥ 5 ng/mL had a significantly lower post-CCRT Hb level than those with pre-CCRT CEA < 5 ng/mL (12.3 g/dL vs. 13.1 g/dL, respectively; *p* = 0.008). This finding provides insight into a potential interaction between CEA and Hb.

**Figure 2:**
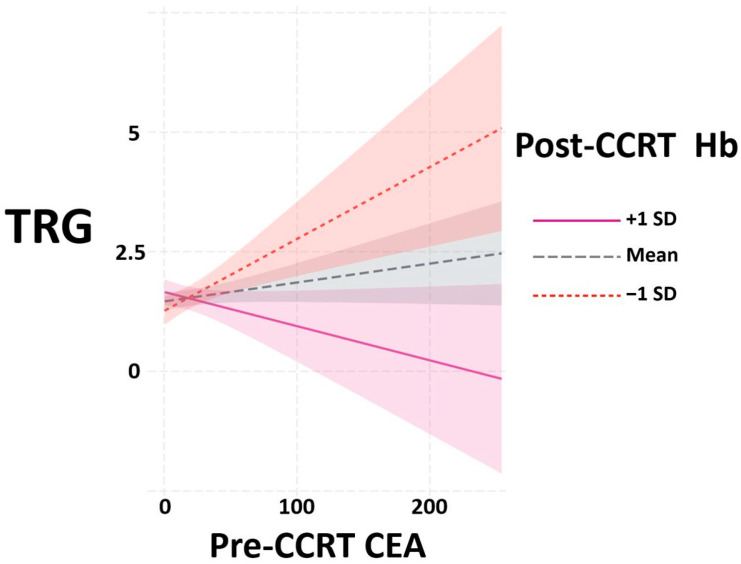
Interaction plots between pre-CCRT CEA and post-CCRT Hb to TRG from R software version 4.2.2. Abbreviations: CCRT, concurrent chemoradiotherapy; CEA, carcinoembryonic antigen; Hb, hemoglobin; TRG, tumor regression grade; SD, standard deviation.

Therefore, we aimed to confirm the impact of Hb and CEA on TRG in patients with rectal cancer. We started by investigating the potential interaction between these two factors. The interaction plots from R software version 4.2.2 in Figure 2 indicate that an interaction existed between pre-CCRT CEA and post-CCRT Hb for TRG outcome. The *x*-axis represents pre-CCRT CEA level, while the *y*-axis represents tumor regression grade. Different line colors, dotted lines and dashed lines represent various levels of post-CCRT Hb. Our findings indicate that in patients with Hb ≥ one standard deviation above the mean (purple line), higher CEA was associated with increased TRG, suggesting a poorer response to CCRT. In patients with Hb ≤ one standard deviation below the mean (red dotted line), the association between pre-CCRT CEA and the outcome variable was the opposite, indicating a positive response to CCRT. This indicates that different Hb levels are associated with opposite correlations between CEA and TRG, suggesting the presence of an interaction between these factors. 

We discovered an interaction effect between post-CCRT Hb and pre-CCRT CEA on TRG, which prompted us to further investigate this relationship. Given that Hb levels differ between males and females, it is essential to conduct separate analyses based on sex to gain a comprehensive understanding of this association. In males, anemia was characterized by Hb levels < 13 g/dL, while for females, anemia was identified by Hb levels < 12 g/dL. CEA was classified as elevated if it was greater than 5 ng/mL. Based on these thresholds, the patients were categorized into two distinct groups: the anemia group and the nonanemia group. We then performed a two-way analysis of variance (ANOVA) using post-CCRT Hb and pre-CCRT CEA as independent variables. This analysis was conducted separately for each sex group (Table 5). In the male group, neither pre-CCRT CEA (F = 0.001, *p* = 0.977) nor anemia (F = 1.744, *p* = 0.192) had a significant main effect on TRG outcomes in men. However, the interaction effect between the pre-CCRT CEA and post-CCRT Hb concentrations was found to be significant (F = 4.376, *p* = 0.041), suggesting the presence of an association between CEA and anemia for TRG outcomes. For the results in the female group, the main effect of pre-CCRT CEA was significant (F = 11.812, *p* = 0.002), while the main effect of post-CCRT Hb was not significant (F = 0.295, *p* = 0.591). Additionally, the interaction effect between pre-CCRT CEA and post-CCRT Hb was significant (F = 5.030, *p* = 0.034), suggesting that the relationship between these variables and TRG is not independent.

**Figure 3:**
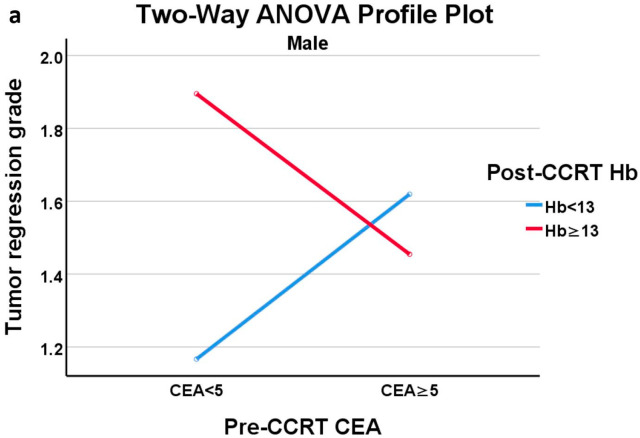

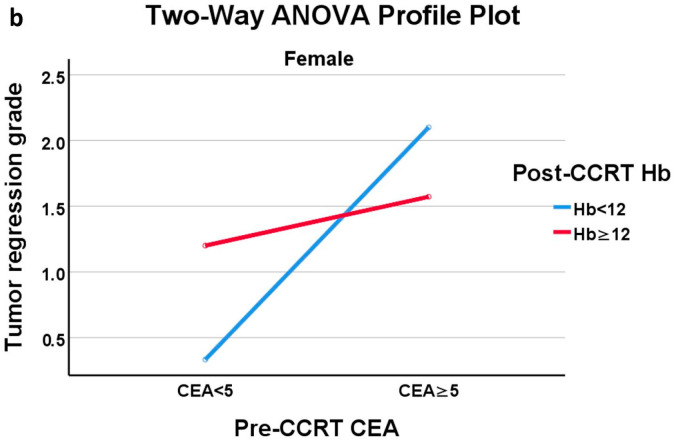
(**a**) Profile plot of two-way ANOVA of male group; (**b**) Profile plot of two-way ANOVA of female group.

In the profile plots of ANOVA in males and females, the crossing of two lines indicated the presence of an interaction effect between pre-CCRT CEA level and the presence of anemia on TRG (Figure 3a,b). Notably, in male patients with normal CEA, the presence of anemia-after-CCRT was linked to a favorable response to CCRT. In female patients, those with normal pre-CCRT CEA showed a more favorable response to CCRT, as evidenced by a lower TRG score, whose effect became more pronounced when the patient also had anemia after CCRT. Further subgroup analysis was conducted using one-way ANOVA with CEA and Hb stratification to assess the impact of anemia-after-CCRT or the pre-CCRT CEA level on TRG. The results (Tables S1 and S2) are consistent with our other findings. Anemia after CCRT had a significant effect on TRG in males with normal CEA levels, and pre-CCRT CEA had a significant effect on TRG in females with anemia present after CCRT.

**Figure 4:**
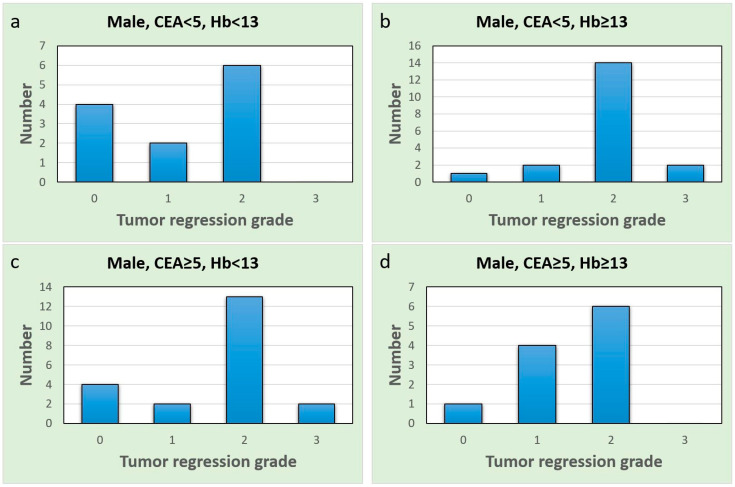
Bar charts of the male group; the *x*-axis represents the tumor regression grade level, and the *y*-axis represents the case number. (**a**) Normal CEA and anemia group; (**b**) normal CEA and non-anemia group; (**c**) elevated CEA and anemia group; (**d**) elevated CEA and non-anemia group.

**Figure 5:**
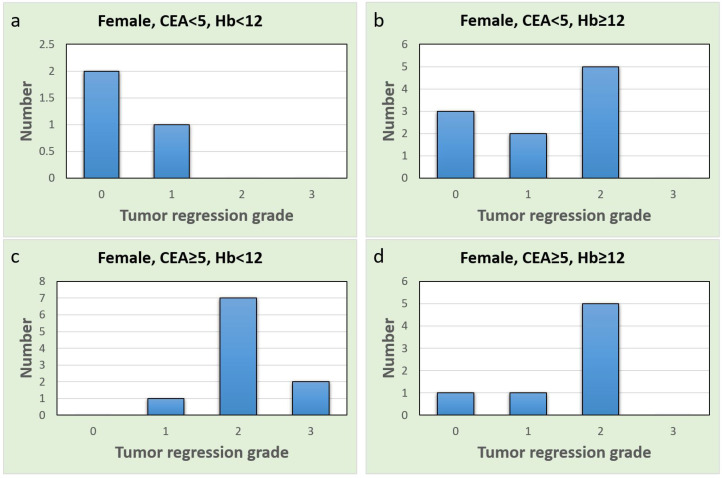
Bar charts of the female group, *x*-axis represents tumor regression grade level, and *y*-axis represents case number (**a**) Normal CEA and anemia group; (**b**) Normal CEA and non-anemia group; (**c**) Elevated CEA and anemia group; (**d**) Elevated CEA and non-anemia group.

These findings highlight the potential sex-specific nature of CEA as a predictive biomarker in the context of our study. A comprehensive discussion of these contradictory and similar findings and their implications will be provided in the next section. To further explore the different subgroups, we drew bar charts (Figure 4 and Figure 5). Upon comparing Figure 4a with Figure 4b, the proportion of complete responses (TRG = 0) and good responses (TRG = 1) was notably higher in males with normal CEA and anemia (Figure 4a). Similar results were observed in females as well (Figure 5a,b). All females in the normal CEA and anemia-after-CCRT subgroup exhibited at least a good response (Figure 5a). In patients with normal CEA levels, the presence of anemia seems to predict a stronger CCRT effect and a favorable CCRT response. Additionally, females in the group with post-CCRT Hb < 12 g/dL and elevated pre-CCRT CEA (Figure 5c) showed no instances of pCR and several poor CCRT responses. In females with anemia, an elevated CEA level indicated a poor response to CCRT.

## 4. Discussion

Our study is the first to examine and elucidate the interaction between clinical factors in predicting the pre-CCRT response of LARC. We observed different post-CCRT Hb levels between different pre-CCRT CEA groups. Then, an interaction effect between pre-CCRT CEA and post-CCRT Hb on TRG outcomes was discovered and verified in different ways.

While no previous studies have specifically investigated the interaction between CEA and Hb in LARC, separate studies have examined the roles of CEA and Hb individually. Studies have suggested a strong association between the Hb level and the response to neoadjuvant CCRT, as well as OS- and LARC-specific survival in elderly patients with rectal cancer [19]. Lee et al. [18] demonstrated the significance of sex and anemia as predictors of pCR through both univariate and multivariate analyses. Additionally, anemia has been associated with poorer DFS. Bong et al. [17] identified anemia and elevated CEA (>5) as factors associated with a lower probability of achieving pCR, although no significant associations were observed with DFS or OS. Anemia typically leads to inadequate tissue oxygenation, compromising the efficacy of radiation therapy. By contrast, we found an interesting association between anemia and treatment response, especially in men belonging to the normal pretreatment CEA group. Intriguingly, posttreatment anemia was linked to improved tumor response in men with normal pre-CCRT CEA, as evidenced by lower tumor regression grade scores. This finding challenges the conventional understanding and highlights the complexity of the treatment response of rectal cancer patients. Unlike previous studies that collected Hb data before or during neoadjuvant CCRT, our study specifically focused on post-CCRT Hb levels. This difference in the timing of Hb assessment may help explain the divergent findings. Anemia, which is a common adverse effect of FOLFOX or 5-FU regimens, could be a contributing factor. The presence of post-CCRT anemia, indicating a more intensive treatment approach, may correlate with improved treatment outcomes, leading to enhanced tumor regression. 

The CEA level has been widely investigated as a predictive biomarker for tumor regression, DFS, and OS in rectal cancer. Cai et al. [20] conducted a study involving 284 patients with elevated pre-CCRT CEA levels and found a correlation between the reduction rate of CEA and tumor regression, as well as downstaging. Additionally, higher post-CCRT CEA levels were predictive of poor OS. Other studies, such as those by Zhao et al. [28] and Lu et al. [21] successfully developed scoring systems by combining CEA with other clinical factors, such as CA-199 or nutritional indices, to predict DFS and OS outcomes. These studies collectively underscore the importance of CEA as a prognostic indicator for evaluating treatment response and long-term outcomes in patients with rectal cancer. Our study revealed that higher pre-CCRT CEA correlated with poorer T downstaging. Further subgroup analysis revealed that women with elevated pre-CCRT CEA and post-CCRT anemia had a poorer tumor responses. These findings underscore the significance of CEA as a prognostic indicator and the potential impact of anemia and its interaction with CEA on treatment outcomes in LARC.

As for other hematological biomarkers, Policicchio et al. [29] reported that patients with elevated platelet and neutrophil counts during diagnosis had a reduced likelihood of achieving pCR with neoadjuvant treatment in LARC. Consistent with this, our findings demonstrate that elevated pretreatment or posttreatment neutrophil counts are associated with poor tumor responses. However, in our investigation, we noted a significant correlation between unfavorable tumor responses and the post-CCRT platelet level, while the pre-CCRT platelet level did not exhibit such an association. This discrepancy in findings regarding platelet counts may be attributed to differences in the criteria used to define the patient groups. Policicchio et al. utilized a cutoff point of 350 platelets in their analysis, which may have influenced the observed associations. Several studies have examined the association between inflammatory indices, such as the P/L ratio and N/L ratio, and clinical outcomes in rectal cancer patients [30,31,32,33,34]. While most of these studies have focused on DFS and OS, there have been some results related to tumor response. Caputo et al. [30] reported that a higher post-CCRT N/L ratio (above the cutoff point of 3.8) was associated with poor tumor regression and increased surgical complications. Similarly, Andras et al. [31] identified the N/L ratio as an independent predictor of poor treatment responses in multivariate regression. A higher P/L ratio was also reported as a predictive factor for poor tumor responses in univariate analysis by Jia et al. [34]. We did not observe a significant association of the N/L ratio or P/L ratio with TRG. Notably, our analysis treated these ratios as continuous variables rather than using predefined cutoff points to divide groups. This difference in approach may contribute to the inconsistency between our findings and previous ones. Additionally, we found that the pre-CCRT and post-CCRT WBC and neutrophil counts, post-CCRT platelet count, and pre-CCRT CEA levels showed correlations with CCRT response.

The underlying mechanism of the interaction between the CEA level and the Hb level has not been elucidated. A few studies have examined the connection between CEA and HbA1c. Chung et al. [35] reported an independent positive relationship between CEA and HbA1c. However, their focus was primarily on potential neoplastic proliferation within a hyperglycemic setting rather than a direct association with the Hb level. Additional investigation is necessary to explore the mechanisms and pathways underlying the interaction between CEA and Hb levels in the context of rectal cancer.

In the last few years, technological developments have been rapid and are continuously evolving, particularly the utilization of the Internet of Medical Things (IoMT). Notably, IoMT has made significant strides in surgical practices [36] and extends its impact to enhancing the quality of life in cancer treatment. IoMT shows promise in managing adverse effects, optimizing sleep patterns, and monitoring physical activity, thereby improving patients’ quality of life [37]. Moreover, IoMT has facilitated the establishment of novel prediction models in rectal cancer [38]. Our future work might integrate advanced technologies, combining postoperative pathological findings with liquid biopsy and improved predictions of CCRT responses. A recent study revealed that collagen features of the pathological tissue can predict the immunoscores of CRC patients and further identify CRC patients who could benefit from adjuvant chemotherapy [39]. Efficiently integrating technology and clinical practice holds the potential to advance personalized medicine, providing a promising avenue for further developments in the field.

Strengths and Limitations

Our research is the first to describe the interaction between CEA and Hb in LARC. We employed a machine learning algorithm, specifically the random forest algorithm, which is known for its ability to handle complex interactions and nonlinear relationships within the data. This approach allowed us to identify the biomarkers most strongly correlated with treatment response. Despite these strengths, our study does have several limitations that warrant acknowledgment. First, our study relied on retrospective data collection, which may introduce selection and information bias. Furthermore, missing data were handled through deletion, which may have resulted in the exclusion of valuable information and potential bias. Also, inhomogeneity in treatment modalities was noted, including various radiotherapy techniques such as IMRT, VMAT, and TOMO, along with differences in chemotherapy regimens. Encouragingly, no notable differences were found in tumor coverage rates among these three radiation therapy methods. However, in terms of sparing organs at risk, TOMO showcased superiority [40]. The variations in chemotherapy regimens indeed highlight this concern. FOLFOX, in particular, demonstrated a higher rate of pCR [41]. Expanding the cohort in future investigations would facilitate a more comprehensive understanding of how variations in chemotherapy impact CCRT outcomes. Another limitation is the generalizability of our findings, as our study was conducted at a single institution. The characteristics of our patient population may not be representative of all rectal cancer patients. Future studies should avoid these limitations and aim to test our findings in larger, prospective cohorts to further enhance their clinical utility and applicability.

## 5. Conclusions

Our study revealed a significant interaction effect between pre-CCRT CEA and post-CCRT Hb levels affecting TRG outcomes, particularly within specific sex-based subgroups. These findings emphasize the importance of considering both CEA and Hb levels, as well as sex, when predicting treatment responses in individuals with rectal cancer who are undergoing neoadjuvant CCRT. Furthermore, our study identified inflammatory biomarkers such as WBC count, neutrophil count, and platelet count as potential predictors of tumor response. Understanding the intricate relationships between CEA, Hb, and the treatment response in rectal cancer patients will aid in optimizing personalized treatment strategies and improving patient outcomes.

## Figures and Tables

**Table 1:** Patients and tumor characteristics (N = 93).

	Pre-CCRT CEA < 5	Pre-CCRT CEA ≥ 5	*p*-Value
	(N (%))	(N (%))	
Age, M ± SD	60.7 (11.6)	61.2 (12.1)	0.840
Gender			
Female	13 (29.5%)	17 (34.7%)	0.760
Male	31 (70.5%)	32 (65.3%)	
Body weight, M ± SD	66.5 (12.0)	63.3 (13.9)	0.250
Tobacco use (+/−)	14/30	13/36	0.740
Pre-CCRT T stage			
T1	2 (4.55%)	0 (0%)	0.380
T2	9 (20.5%)	6 (12.2%)	
T3	28 (63.6%)	33 (67.3%)	
T4a	4 (9.09%)	8 (16.3%)	
T4b	1 (2.27%)	2 (4.08%)	
Pre-CCRT N stage			
N0	17 (38.6%)	15 (30.6%)	0.250
N1	20 (45.5%)	19 (38.8%)	
N2	7 (15.9%)	15 (30.6%)	
Pre-CCRT Total stage			
I	8 (18.2%)	3 (6.12%)	0.075
IIA	9 (20.5%)	10 (20.4%)	
IIB	0 (0%)	1 (2.04%)	
IIIA	3 (6.82%)	0 (0%)	
IIIB	15 (34.1%)	26 (53.1%)	
IIIC	7 (15.9%)	4 (8.16%)	
IV	2 (4.55%)	5 (10.2%)	
Underlying disease			
Diabetes mellitus (+/−)	32/12	41/8	0.300
Hypertension (+/−)	26/18	27/22	0.860
Hyperlipidemia (+/−)	39/5	46/3	0.470
Chemotherapy regimen			
FOLFOX	16 (36.4%)	20 (40.8%)	0.800
5-FU/LV	24 (54.5%)	26 (53.1%)	
Others	4 (9.09%)	3 (6.12%)	
Oral C/T after CCRT (+/−)	28/16	21/28	0.073

Abbreviations: CCRT, concurrent chemoradiotherapy; M, mean; SD, standard deviation; FOLFOX, folinic acid, fluorouracil, and oxaliplatin; 5-FU/LV, fluorouracil and leucovorin; C/T, chemotherapy.

**Table 2:** Univariate analysis of tumor response.

	TRG		
	Favorable Response	Unfavorable Response	*p*-Value
	(N = 32) Mean (SD)	(N = 64) Mean (SD)	
Pre-CCRT CEA (ng/mL)	12.0 (26.6)	22.5 (41.4)	0.200
Post-CCRT CEA (ng/mL)	2.88 (1.50)	8.28 (26.2)	0.260
CEA reduction rate	0.726 (0.530)	0.597 (0.505)	0.260
Pre-CCRT Hb (g/dL)	12.8 (2.19)	12.3 (2.32)	0.380
Post-CCRT Hb (g/dL)	12.7 (1.60)	12.7 (1.44)	0.930
Pre-CCRT WBC (10^3^/μL)	6.95 (1.84)	8.07 (2.34)	0.020
Post-CCRT WBC (10^3^/μL)	4.82 (1.47)	5.87 (1.53)	0.002
Pre-CCRT neutrophil (10^3^/μL)	4.39 (1.52)	5.37 (2.36)	0.041
Post-CCRT neutrophil (10^3^/μL)	3.00 (1.04)	3.74 (1.21)	0.083
Pre-CCRT lymphocyte (10^3^/μL)	1.68 (0.583)	1.72 (0.727)	0.800
Post-CCRT lymphocyte (10^3^/μL)	0.678 (0.284)	1.01 (0.440)	0.025
Pre-CCRT platelet (10^3^/μL)	267 (90.4)	284 (90.7)	0.390
Post-CCRT platelet (10^3^/μL)	177 (49.2)	230 (64.4)	<0.001
Pre-CCRT N/L ratio	2.73 (1.23)	3.86 (3.77)	0.120
Post-CCRT N/L ratio	4.98 (2.60)	4.55 (2.70)	0.650
Pre-CCRT P/L ratio	174 (87.2)	190 (123)	0.510
Post-CCRT P/L ratio	270 (90.5)	265 (149)	0.920

Favorable response: tumor regression grade = 0 or 1; unfavorable response: tumor regression grade = 2 or 3. Abbreviations: CCRT, concurrent chemoradiotherapy; CEA, carcinoembryonic antigen; Hb, hemoglobin; WBC, white blood cells; N/L, neutrophil to lymphocyte; P/L, platelet to lymphocyte.

**Table 3:** Univariate analysis of tumor down stage.

	T Stage				
	Complete Response	Good Response	Stable Disease	Disease Progression	*p*-Value
	(N = 16) Mean (SD)	(N = 27) Mean (SD)	(N = 45) Mean (SD)	(N = 8) Mean (SD)	
Pre-CCRT CEA (ng/mL)	9.81 (24.2)	11.4 (24.5)	20.3 (27.9)	60.2 (96.8)	0.012
Post-CCRT CEA (ng/mL)	3.00 (1.56)	2.75 (2.40)	10.1 (31.1)	6.43 (6.64)	0.490
CEA reduction rate	0.859 (0.465)	0.642 (0.444)	0.585 (0.503)	0.516 (0.848)	0.310
Pre-CCRT Hb (g/dL)	12.2 (2.37)	12.5 (2.44)	12.6 (2.21)	12.2 (2.20)	0.910
Post-CCRT Hb (g/dL)	12.2 (1.56)	13.3 (1.37)	12.5 (1.54)	12.7 (0.898)	0.077
Pre-CCRT WBC (10^3^/μL)	6.63 (1.83)	8.64 (2.91)	7.60 (1.81)	7.24 (1.64)	0.029
Post-CCRT WBC (10^3^/μL)	4.45 (1.36)	5.68 (1.57)	5.74 (1.53)	5.89 (1.75)	0.027
Pre-CCRT neutrophil (10^3^/μL)	4.24 (1.65)	6.01 (3.20)	4.83 (1.43)	4.56 (1.25)	0.049
Post-CCRT neutrophil (10^3^/μL)	2.65 (1.10)	3.52 (1.39)	3.79 (0.983)	3.90 (1.50)	0.170
Pre-CCRT lymphocyte (10^3^/μL)	1.61 (0.660)	1.66 (0.781)	1.75 (0.642)	1.74 (0.654)	0.880
Post-CCRT lymphocyte(10^3^/μL)	0.601 (0.140)	1.02 (0.389)	0.800 (0.286)	1.77 (0.159)	<0.001
Pre-CCRT platelet (10^3^/μL)	282 (110)	302 (107)	274 (71.2)	216 (62.0)	0.120
Post-CCRT platelet (10^3^/μL)	184 (58.8)	221 (66.3)	215 (63.8)	225 (68.4)	0.260
Pre-CCRT N/L ratio	2.70 (1.38)	4.65 (5.48)	3.21 (1.77)	2.90 (1.27)	0.210
Post-CCRT N/L ratio	4.80 (2.79)	4.02 (2.30)	5.49 (2.73)	2.27 (1.10)	0.130
Pre-CCRT P/L ratio	191 (105)	212 (165)	177 (79.5)	127 (45.5)	0.320
Post-CCRT P/L ratio	301 (83.3)	257 (136)	287 (149)	141 (68.1)	0.220

Complete response: no residual tumor; good response: T down stage; stable disease: no change of T stage; disease progression: elevated T stage. Abbreviations: CCRT, concurrent chemoradiotherapy; CEA, carcinoembryonic antigen; Hb, hemoglobin; WBC, white blood cells; N/L, neutrophil to lymphocyte; P/L, platelet to lymphocyte.

**Table 4:** Univariate analysis after CCRT.

	Pre-CCRT CEA < 5	Pre-CCRT CEA ≥ 5	*p*-Value
	(N = 44) Mean (SD)	(N = 49) Mean (SD)	
Post-CCRT Hb (g/dL)	13.1 (1.44)	12.3 (1.48)	0.008
Post-CCRT WBC (10^3^/μL)	5.27 (1.46)	5.66 (1.66)	0.230
Post-CCRT neutrophil (10^3^/μL)	3.38 (1.27)	3.69 (1.16)	0.440
Post-CCRT lymphocyte (10^3^/μL)	0.807 (0.350)	0.968 (0.432)	0.220
Post-CCRT platelet (10^3^/μL)	205 (58.8)	220 (70.9)	0.280
Post-CCRT N/L ratio	4.80 (2.51)	4.73 (2.80)	0.930
Post-CCRT P/L ratio	270 (105)	273 (157)	0.950

Abbreviations: CCRT, concurrent chemoradiotherapy; CEA, carcinoembryonic antigen; Hb, hemoglobin; WBC, white blood cells; N/L, neutrophil to lymphocyte; P/L, platelet to lymphocyte.

**Table 5:** Two-way ANOVA results for the association between pre-CCRT CEA levels, post-CCRT hemoglobin concentration, and TRG in patients with rectal cancer in male/female.

Dependent Variable: Tumor Regression Grade (TRG)						
	Source	Type III Sum of Squares	df	Mean Square	F Value	Sig.
Male	Pre-CCRT CEA	0.001	1	0.001	0.001	0.977
	Post-CCRT Hb	1.157	1	1.157	1.744	0.192
	CEA * Hb	2.902	1	2.902	4.376	0.041
Female	Pre-CCRT CEA	6.761	1	6.761	11.812	0.002
	Post-CCRT Hb	0.169	1	.169	0.295	0.591
	CEA * Hb	2.879	1	2.879	5.030	0.034

Abbreviations: CCRT, concurrent chemoradiotherapy; CEA, carcinoembryonic antigen; Hb, hemoglobin; *, interaction between two factors.

## Data Availability

The datasets used and/or analyzed during the current study are available from the corresponding author upon request.

## Supplementary Materials

The following supporting information can be downloaded at: https://www.mdpi.com/article/10.3390/jcm12247690/s1, Table S1: ANOVA tests in subgroups of sex and CEA; Table S2: ANOVA tests in subgroups of sex and Hb level.
