# Supplementary material for: Predictive Value of the Interaction between CEA and Hemoglobin in Neoadjuvant CCRT Outcomes in Rectal Cancer Patients
Source: J Clin Med. 2023 Dec 14;12(24):7690. doi: 10.3390/jcm12247690 (PMC10744245; doi:10.3390/jcm12247690)
Supplement: Supplementary file 1 [file jcm-12-07690-s001.zip › jcm-2673793-supplementary.pdf]

Table S1: ANOVA tests in subgroups of sex and CEA.

ANOVA of Hb and TRG							
**TRG**	**Groups: anemia or not**		**SS**	**df**	**Mean Square**	**F**	**Sig.**
Male	CEA<=5	Between Groups	3.899	1	3.899	6.477	.017
		Within Groups	17.456	29	.602		
	CEA>5	Between Groups	.195	1	.195	.270	.607
		Within Groups	21.680	30	.723		
Female	CEA<=5	Between Groups	1.733	1	1.733	2.306	.157
		Within Groups	8.267	11	.752		
	CEA>5	Between Groups	1.150	1	1.150	2.609	.127
		Within Groups	6.614	15	.441		

Dependent variable: tumor regression grade

Abbreviations: TRG, tumor regression grade; CEA, carcinoembryonic antigen

Table S2: ANOVA tests in subgroups of sex and Hb level

ANOVA of CEA and TRG							
**TRG**	**Groups: elevated CEA or not**		**SS**	**df**	**Mean Square**	**F**	**Sig.**
Male	Hb<13	Between Groups	1.563	1	1.563	1.820	.187
		Within Groups	26.619	31	.859		
	Hb>=13	Between Groups	1.350	1	1.350	3.020	.093
		Within Groups	12.517	28	.447		
Female	Hb<12	Between Groups	7.203	1	7.203	22.214	.001
		Within Groups	3.567	11	.324		
	Hb>=12	Between Groups	.568	1	.568	.753	.399
		Within Groups	11.314	15	.754		

Dependent variable: tumor regression grade

Abbreviations: TRG, tumor regression grade; Hb, Hemoglobin
